# Management of Recurrent Implantation Failure and Hereditary Thrombophilia: A Case Report

**DOI:** 10.7759/cureus.52882

**Published:** 2024-01-24

**Authors:** Tamar Barbakadze, Tengiz Zhorzholadze, Nino Kutchukhidze, Mariam Shervashidze, Tea Charkviani

**Affiliations:** 1 Gynecology, ReproArt Georgian-American Center for Reproductive Medicine, Tbilisi, GEO; 2 Genetics, ReproArt Georgian-American Center for Reproductive Medicine, Tbilisi, GEO; 3 Obstetrics and Gynecology, ReproArt Georgian-American Center for Reproductive Medicine, Tbilisi, GEO

**Keywords:** preimplantation genetic testing for aneuploidy (pgt-a), hereditary thrombophilia, oocyte donation, aging of the endometrium, endometrial receptivity analysis (era), recurrent implantation failure (rif)

## Abstract

Recurrent implantation failure (RIF) is one of the core problems for assisted reproductive technology (ART). High-quality, euploid embryos and synchronization between the embryonic stage and the uterine endometrial lining are crucial for positive outcomes. Molecular biology techniques have significantly transformed assisted reproductive technology (ART). Numerous couples facing infertility issues have successfully achieved the birth of healthy infants through the application of molecular biology methods: preimplantation genetic testing for aneuploidy (PGT-A) and endometrial receptivity analysis (ERA). Exploring the impact of age on endometrial assays like the endometrial receptivity assay (ERA) yields valuable insights, including the determination of the implantation window and the development of personalized strategies.

The authors present the case of a 42-year-old woman who has experienced RIF with euploid embryos, coupled with a hereditary thrombophilia homozygous mutation in the MTHFR genes: A1298C and C677T.

## Introduction

Molecular biological techniques have brought a significant transformation in assisted reproduction. Numerous couples facing infertility issues have successfully achieved the birth of healthy infants thanks to applying two molecular biology methods: preimplantation genetic testing for aneuploidy (PGT-A) [1] and endometrial receptivity analysis (ERA) [2].

The fact that the oocytes play a crucial role in reproductive ageing in women is quite noteworthy. However, as long as the endometrium is pivotal in facilitating embryo implantation and subsequent pregnancy, exploring the impact of age on endometrial assays, like the ERA, provides valuable insights as well.

This underscores a critical facet of age-related fertility decline that extends beyond ovarian considerations. While advancements in assisted reproductive technologies (ARTs), including oocyte donation and PGT-A, have effectively addressed specific challenges associated with ovarian ageing, there is a growing acknowledgement that other factors, particularly the ageing of the endometrium, exert a substantial influence on reproductive outcomes for women of advanced age. With advancing age, alterations in the endometrium may transpire, potentially impacting implantation rates, clinical pregnancy, and live births.

Examining these effects within the in-vitro fertilization (IVF) studies involving oocyte donation cycles offers valuable insights for clinicians and researchers in customizing fertility treatments. It underscores the necessity for a comprehensive approach to assisted reproduction, taking into account both ovarian and endometrial factors to optimize outcomes for women undergoing fertility treatments later in life [3].

## Case presentation

A female patient, aged 42, sought infertility treatment. The patient received a diagnosis of secondary infertility due to the diminished ovarian reserve resulting from her advanced age. In her medical history, the initial pregnancy occurred at the age of 21, complicated by preeclampsia. Throughout this initial pregnancy, a healthy baby was delivered via cesarean section (CS). Given the history of preeclampsia, a test was conducted to rule out hereditary thrombophilia to mitigate obstetric complications. The results revealed a homozygous mutation in the MTHFR genes: A1298C and C677T. When she visited our clinic 21 years later, she reported a history of two years of secondary infertility, including an unsuccessful autologous IVF cycle with the transfer of a single embryo. The initial testing diagnosed the patient with adenomyosis. An office hysteroscopy was utilized for uterine cavity assessment.

Under our care, she underwent a donor oocyte cycle, primarily due to her age and limited ovarian reserve. Her partner was 50 years old. The semen parameters were the following: concentration (106 per ejaculate)38 x 10^6 ml (according to WHO recommendations, normal ≥ 39 x 10^6 ml); total motility 27% (according to WHO recommendations, normal ≥ 42%); progressive motility 40% (according to WHO recommendations, normal ≥ 30%); and morphology 1% (according to WHO recommendations, normal ≥ 4%). The egg donor signed the consent form conferring the authority for decision-making regarding unused embryos to the designated couple. The 22-year-old egg donor presented with an antral follicle count (AFC) level of 34 and an anti‑Mullerian hormone (AMH) level of 5.4 ng/mL. The controlled ovarian stimulation for the egg donor was done using 375 IU recombinant follicle-stimulating hormone (FSH), with 75 IU human menopausal gonadotropin (HMG) for the first two days, followed by 150 IU recombinant FSH and 75 IU HMG from the third day of ovarian stimulation. As for the trigger, gonadotropin hormone-releasing hormone (GnRH) agonist 0.2mg/2ml and human chorionic gonadotropin (HCG) 1500 IU were used. The duration of ovarian stimulation was 12 days, resulting in the retrieval of 19 oocytes, of which 17 were in the metaphase II (MII) stage. Intracytoplasmic sperm injection (ICSI) was performed, yielding 14 fertilized embryos. On the fifth day, a trophectoderm biopsy was conducted on four blastocysts, which were subsequently frozen. On the sixth day, an additional four blastocysts were obtained. PGT-A was performed during this process, utilizing next-generation sequencing (NGS). Out of the eight blastocysts assessed, four were identified as euploid embryos.

Endometrium for a frozen embryo transfer (FET) was prepared with hormone replacement therapy (HRT), using 6mg oral estradiol and 3gr transdermal estradiol daily. The duration of estradiol days was 14 days; for luteal phase support, vaginal progesterone 600mg and dydrogesterone 20mg per day were used. Endometrial thickness on the day of progesterone introduction was 7.5mm; progesterone level in the blood was measured as 0.1 ng/mL before progesterone introduction; embryo transfer was performed 120 hours after progesterone introduction. We transferred two euploid embryos with grades 3AA and 3AA. Even though two high-grade euploid embryos had been transferred in a FET cycle, pregnancy did not occur.

After this, a timed endometrial biopsy was conducted and sent for ERA. Endometrium for endometrial biopsy was prepared with HRT- 6mg oral estradiol and 3gr transdermal estradiol per day. The duration of estradiol days was 12 days. For luteal phase support, vaginal progesterone 600mg and dydrogesterone 20mg per day were used. The endometrial thickness on the day of progesterone introduction was 7.6mm; the progesterone level in the blood was measured as 0.1 ng/mL before progesterone introduction. An endometrial biopsy was conducted 120 hours after the initiation of progesterone. The results from the ERA suggested that a personalized embryo transfer (ET) should occur within a window of 145±3 hours after the commencement of progesterone. The endometrium for the embryo transfer was primed using HRT involving 6mg of oral estradiol and 3gr of transdermal estradiol per day, with a 14-day duration of estradiol administration. For luteal phase support, 600mg of vaginal progesterone and 20mg of dydrogesterone were administered daily. The endometrial thickness on the day of progesterone initiation measured 7.5mm, with a pre-introduction blood progesterone level of 0.1 ng/mL. Two euploid embryos, with qualities of 3CB and 3BC, respectively, were transferred after 145 hours from the commencement of progesterone. Beginning from the day of embryo transfer, the patient received a daily injection of enoxaparin sodium at a dosage of 40mg/0.4 ml. Additionally, she took L-methyl folate at a dose of 200mcg and folic acid at a dose of 200mcg each day.

The subsequent ET was meticulously timed based on the ERA findings, resulting in a slight deviation of 24 hours from our standard practice. During this procedure, two euploid embryos were successfully transferred. A positive β-hCG test was observed 12 days after the ET. At the six-week mark, an ultrasound revealed the presence of one amniotic sac and one embryo. The pregnancy progressed smoothly despite the ET being conducted 24 hours later than our usual FET timing. Ultimately, at 39 weeks, a healthy female newborn was delivered via CS without complications.

## Discussion

The impact of advanced maternal age on the reproductive system encompasses several changes, and these alterations can significantly influence cellular senescence and endometrial receptivity, thereby contributing to infertility.

In the endometrium context, advanced maternal age may be associated with compromised cellular senescence, adversely affecting endometrial cells' general health and functionality. Factors including fluctuations in hormonal levels, modifications in the extracellular matrix of the endometrium, and compromised blood flow to the uterus can collectively contribute to this impaired receptivity [3]. Consequently, this impairment of endometrial receptivity influences the uterine lining's ability to support embryo implantation, diminishing the likelihood of a successful pregnancy.

Molecular biological techniques such as PGT-A and ERA can significantly improve the outcome. PGT-A in the donor egg cycle improves the implantation rate, decreases the spontaneous abortion rate, and increases the ongoing pregnancy/live birth rate. At the same time, ERA gives the ability to formulate personalized strategies and interventions for the patient.

In our case report, the recurrent implantation failure, despite transferring the high-grade euploid embryos, made it clear that standard FET protocol was impractical, so a personalized embryo transfer was performed based on PGT-A and ERA findings.

As mentioned earlier, the patient was diagnosed with hereditary thrombophilia homozygous mutation of the MTHFR genes: A1298C and C677T, which is associated with pregnancy complications like preeclampsia, recurrent pregnancy loss, fetal growth retardation, etc. So, despite the contra version's beneficial effect of 5-methylenetetrahydrofolate, we believe the supplementation with 5-methylenetetrahydrofolate helped improve our case's reproductive outcomes. From the day of embryo transfer, enoxaparin sodium was given daily.

Ultimately, this case report underscores the efficacy of the combined utilization of PGT-A and ERA techniques in attaining live births in cases of patients with complicated medical histories (advanced age, hereditary thrombophilia homozygous mutation of the MTHFR genes, and RIF) [4,5].

## Conclusions

Our case report of a live birth achieved through the combined utilization of PGT-A and ERA underscores the efficacy of molecular biological techniques in attaining live births, even in cases involving patients with complicated medical histories. This demonstrates that cutting-edge technology can be successfully employed in less affluent countries where IVF remains economically accessible.

The presented single case report provides a promising foundation. However, future studies must generate enough evidence to fill the existing knowledge gap and justify the widespread utilization of ERA in combination with PGT-A in managing patients with RIF.

Acknowledging the influence of advanced maternal age not only on ovarian reserve but also on the cellular and tissue-level changes of endometrium holds paramount importance for fertility specialists. Examining these effects within the IVF studies involving oocyte donation cycles offers valuable insights for clinicians and researchers in customizing fertility treatments. It underscores the necessity for a comprehensive approach to assisted reproduction, considering both ovarian and endometrial factors to improve the prospects of a successful pregnancy for women facing age-related fertility challenges.
